# Spray-Dried Proliposomes: an Innovative Method for Encapsulation of *Rosmarinus officinalis* L. Polyphenols

**DOI:** 10.1208/s12249-020-01668-2

**Published:** 2020-05-18

**Authors:** Victor Oloruntoba Bankole, Michael Oluwole Osungunna, Claudia Regina Fernandes Souza, Sergio Luiz Salvador, Wanderley Pereira Oliveira

**Affiliations:** 1 Faculty of Pharmaceutical Sciences of Ribeirão Preto, University of São Paulo, Av. do Café s/n, Ribeirão Preto, SP 14040-903 Brazil; 2 Department of Pharmaceutics, Faculty of Pharmacy, Obafemi Awolowo University, Ile-Ife, 220005 Nigeria

**Keywords:** rosemary polyphenols, proliposomes, spray drying, design of experiment, antioxidant activity, antifungal activity

## Abstract

This work aims to improve the functionality of *Rosmarinus officinalis* L. (rosemary) polyphenols by encapsulation in an optimized proliposome formulation. A 2^3^ Box-Wilson central composite design (CCD) was employed to determine lone and interaction effects of composition variables on moisture content (*X*_p_); water activity (*A*_w_); concentration and retention of rosemary polyphenols—rosmarinic acid (ROA), carnosol (CAR), and carnosic acid (CNA); and recovery of spray-dried proliposomes (SDP). Processing conditions which generate proliposomes with optimum physicochemical properties were determined by multi-response analysis (desirability approach). Antioxidant and antifungal activities were evaluated by 1,1-diphenyl-2-picrylhydrazyl (DPPH^•^) sequestering and minimum inhibitory concentration (MIC)/minimum fungicidal concentration (MFC) assays, respectively. SDP exhibited high polyphenol retention, ranging from 62.0 to 100.0% w/w, showing dependence on composition variables and polyphenol lipophilicity. SDP recovery ranged from 20.1 to 45.8%, with *X*_p_ and *A*_w_ of 1.7 ± 0.14–2.5 ± 0.23% w/w and 0.30 ± 0.004–0.47 ± 0.003, respectively, evidencing product with good chemical and microbiological stability. Optimum liposomal composition was determined, namely, lipid concentration (4.26% w/w), lyophilized extract (LE) concentration (4.48% w/w), and drying aid:(lipid+extract) ratio (7.55% w/w) on wet basis. Relative errors between experimental and predicted values for SDP properties showed concurrence for all responses except CAR retention, being 22% lower. SDP showed high antioxidant activity with IC_50_ of 9.2 ± 0.2 μg/mL, superior to results obtained for LE (10.8 μg/mL) and butylated hydroxytoluene (BHT), a synthetic antioxidant (12.5 μg/mL). MIC and MFC against *Candida albicans* (ATCC1023) were 312.5 μg/mL and 1250 μg/mL, respectively, a moderate antimicrobial activity for phytochemical-based products. SDP is shown as a veritable tool to encapsulate hydrophilic and lipophilic rosemary polyphenols generating a product with optimal physicochemical and biological properties.

## INTRODUCTION

Currently, plant polyphenols have received high attention of the pharmaceutical, nutraceutical, cosmetic, and food sectors mainly due to their attributed biological activities. *Rosmarinus officinalis* L., commonly called rosemary, is an aromatic herbaceous plant from the Lamiaceae family, native to the Mediterranean region, but cultivated all over the world. It is widely used as food flavoring and preservative and as herbal drug in folk medicine (1–3).

The rosemary is a polyphenol-rich herb, supporting its use as preservative and antioxidant in cosmetics, foods, and other multi-component systems, as well as herbal remedy in protection from and management of various degenerative diseases associated with oxidative stress including cancers, cardiovascular diseases, neurodegenerative diseases, diabetes, age-related skin damage, and osteoporosis (4–6). Indeed, the rosemary extracts are commercially available for use as a natural antioxidant for foods, being considered safe and effective (7). The biological activities of rosemary are linked to the presence of phenolic constituents pertaining to three main classes: phenolic diterpenes (e.g., carnosic acid, carnosol, rosmanol, epirosmanol, and methyl carnosate), flavonoids (e.g., cirsimaritin, genkwanin), and phenolic acids (e.g., rosmarinic and caffeic acids), besides others (1,8–13).

In general, the composition and other properties of a plant extract is significantly affected by the extraction procedure (e.g., solvent type, extraction method, time, temperature), which could be optimized to extract the maximum amount of desired substances; rosemary polyphenols in the present work. The high scavenging properties towards radical oxygen exhibited by the rosemary polyphenols and extracts make them susceptible to degradation reactions during storage due to several factors such as heat, humidity exposure, and processing conditions, strongly reducing their long-term stability (12,14–16). CNA is the phenolic diterpene found in higher concentration in the green rosemary leaves which, together with CAR, account for about 90% of its antioxidant activity (17). Moreover, the rosemary antioxidants are highly lipophilic, which hinder their use in aqueous systems.

Encapsulation of plant extracts in different materials is a credible way to improve their physicochemical properties and to slow down the degradation rates of their main active constituents. The improvement of bioavailability of the bioactive compounds in biological systems has also been reported (18,19). A literature review shows several reports on the encapsulation of rosemary polyphenols in solid lipid nanoparticles (20,21) focusing primarily on the encapsulation of CNA rather than various compounds (18). These previously developed systems have as major limitation the inefficiency in accommodating lipophilic and hydrophilic compounds together, which might impair product activity and stability. However, pharmaceutical and cosmetic systems are generally complex in nature, and most often require the simultaneous incorporation of multiple bioactive compounds due to synergism between the constituents. Proliposome is an innovative approach capable of encapsulating variable lipo-hydrophilicity compounds into a single structure. This methodology has been employed in the formulation of different compounds of natural origin (22–26). Proliposomes are free-flowing dry powders developed from phospholipids, usually in conjunction with cholesterol and other excipients.

Liposome suspensions can easily be formed when needed, through the simple redispersion of these systems in water (27). Their dry solid properties improve the otherwise challenging physical stability of liposomes without influencing their intrinsic characteristics (28). An attempt to encapsulate plant polyphenols in this type of structure is therefore attractive for two main considerations. First of all, variable polarity compounds can be entrapped simultaneously to the liposome system. Although the hydrophilic core provides a suitable environment for polar compounds, the liposome wall lipid composition can be exploited to encapsulate more lipophilic polyphenol compounds (29). Secondly, this encapsulation approach can provide protection for bioactive components and increase their solubility and functionality (29,30). These characteristics are particularly desirable for polyphenols that have high radical oxygen scavenging properties, a characteristic that is unfortunately also linked to their lack of long-term stability (14,16). In addition, polyphenols generally have low water solubility and low bioavailability (31,32). The astringency and bitter taste exhibited by various polyphenols might also limit their use, for example, in oral medications and products (33–36), thereby justifying the need for encapsulation. Nevertheless, the production of proliposomes by spray drying is a multivariate process. The physicochemical properties of product are affected by composition variables and spray drying operating conditions (37). Understanding the effects of these multiple input (independent) variables on product properties is an important step towards consistently engineering a product with preset requirements (38–40). The design of experiment (DoE) is an efficient methodology usually adopted to determine the effects of multiple variables on product properties. This approach allows for simultaneous variation of all input parameters rather than assessing the effect of each one on desirable outcomes per time, permitting analysis of their individual and interaction effect on measured responses (41–43). DoE permits the use of statistical tools such as response surface methodology (RSM) for a rapid, cost effective, and accurate assessment of effects towards optimizing the conditions to achieve desirable product qualities and process performance (44,45).

Therefore, the objective of this work was to investigate the effects of composition variables on physicochemical properties of the formed proliposomes and spray drying performance. The goal is to develop an optimized SDP loaded with the main rosemary polyphenols—ROA, CAR, and CNA—in increasing degree of lipophilicity. The antioxidant activity using the DPPH^•^ scavenging method and the antifungal activity against a strain of *Candida albicans* (used as a model) of the optimized SDP were evaluated, to highlight the potential of product application in pharmaceutical, nutraceutical, and cosmetic products.

## EXPERIMENTAL

### Materials

Dried leaves of *Rosmarinus officinalis* L. were acquired from Santos Flora, Mairiporã, São Paulo (LOT: 1505080153). The dried vegetable material was milled in a knife mill (Marconi model MA 680, Brazil) until generated particles pass through a mesh-20 sieve (833 μm). Phospholipon^®^ 90H (hydrogenated soy phosphatidylcholine) was purchased from Lipoid GMBH (Ludwigshafen, Germany). Cholesterol, methanol, and acetonitrile were purchased from Sigma-Aldrich (St Louis, USA). Reference standards of ROA, CAR, and CNA were purchased from Sigma-Aldrich (St Louis, USA). Lactose was purchased from Natural Pharma (Sao Paulo, Brazil). Ethanol was purchased from Labsynth (SP, Brazil). Terbinafine from Fagron (China) was donated by the Pharmacy Education Unit of the Faculty of Pharmaceutical Sciences of Ribeirao Preto, University of Sao Paulo, Brazil. Sabouraud dextrose broth and Sabouraud dextrose agar were purchased from BD Difco™ (USA). Other reagents and solvents used were of analytical grade.

### Preparation and Characterization of Rosemary Extract

The method used to produce the rosemary extract was based on previous studies by our group (46,47). Hydroalcoholic extract of milled vegetable material was prepared by dynamic maceration in a jacketed stirred vessel (under mechanical stirring of 200 rpm) at 70°C for 60 min using 70% v/v ethanol as solvent. The extract obtained was vacuum filtered (filter paper) and concentrated to about 10% solid content by rotary evaporation at 50°C and vacuum pressure of 600 mmHg. Concentrated extract was congealed at – 20°C over 24 h and then placed into a – 80°C ultra-freezer for further 2 h. The congealed sample was freeze-dried in a VLP 195 FD-115, Thermo Fisher Scientific Lyophilizer at condensation temperature of – 40°C for 48 h. The freeze-dried product was placed in airtight amber bottles and stored at − 20°C until use.

#### Quantification of Polyphenol Markers in the Freeze-Dried Rosemary Extract

The biological properties of rosemary are usually linked to the high content of polyphenolic compounds, mainly ROA, CAR, and CNA, and mildly caffeic acid (CFA). The concentrations of these compounds in the freeze-dried extract were determined by HPLC-DAD according to the method proposed by Wellwood and Cole (48), with some modifications. Analyses were performed in a HPLC Shimadzu Prominence LC-20A series with a LC-6A double pump (Shimadzu Corporation, Kyoto, Japan) using a C-18 column (Shimadzu Shim-Pack CLC(M) 4.6 mm × 25 cm, 5 mm, 100 Å) at 30°C. The mobile phase was a gradient of 0.1% formic acid in water (A) and acetonitrile (B). The acetonitrile concentration was varied as formic acid in water (A) and acetonitrile (B). The acetonitrile concentration was varied as follows: 0–20 min, 15–35% B; 20–30 min, 35–100% linear increase of B; 30–35 min, 100% B; and 35–37 min, linear decrease of B to 15%, 37–42 min, 15% B. The chromatograms were recorded at the wavelengths of 284 and 330 nm (49).

The marker compounds were associated with chromatographic peaks corresponding to them by comparing their retention times and spectra with those of analytical grade reference standards. Quantification of CFA, ROA, CAR, and CNA in the LE was performed by integrating the peaks and comparing with those of the external standards, using calibration curves. Samples were filtered through a 0.45-μm Millipore membrane, and 10 μL was injected into the chromatograph. Results are expressed as mean and standard deviation from triplicate assays. The HPLC method was revalidated before use in this work (50).

### Encapsulation of Rosemary Polyphenols in Proliposomes

The proliposome production starts with the encapsulation of the rosemary polyphenols in liposomal compositions, using the solvent replacement method (51) with some modifications. The lipid phase consisted of preset quantities of hydrogenated soy phosphatidylcholine (Phospholipon^®^ 90H) and cholesterol (9:1) dissolved in 50 mL of ethanol 90% v/v at 65°C, while aqueous phase consisted of a dispersion of LE in purified water. The two phases were brought to the same temperature before the lipid phase was injected into the aqueous phase under agitation. Residual alcohol in the system was vacuum removed at 48°C/600 mmHg. The liposomal composition obtained was put to rest at 8°C for 24 h for complete stabilization and then mixed with lactose (drying aid) before submission to spray drying to produce the proliposomes.

The drying runs were carried out in a bench-top SD-05 spray dryer (Lab-Plant UK Ltd., Huddersfield, UK) with a concurrent flow regime, having a drying chamber of 215 mm diameter and 500 mm height. The spray dryer was previously stabilized with the feed of distilled water at same drying conditions. The outlet drying temperature was monitored at regular intervals, when the system attained steady state the feed of liposomal composition (plus lactose) at room temperature (25°C) commenced. The composition was fed at a flowrate of 4 g/min (provided by a peristaltic pump) through a twin fluid atomizer (1.0 mm of orifice diameter) with internal mixing connected to a compressed air line. The flowrate of atomizing air was maintained at 17 L/min at pressure of 1.5 kgf/cm^2^. The inlet gas temperature and flow rate were maintained at 100°C and 60 m^3^/h, respectively. The drying conditions were set according to previous works developed by our group (47). The concentration of solids in the feed was maintained constant at 10.9% w/w for all formulations developed, by using distilled water.

#### Design of Experiments

The effects of three composition variables on physicochemical properties of SDP were studied by using a completely randomized 2^3^ Box-Wilson CCD. The composition variables studied were the total lipid concentration, concentration of rosemary extract, and the drying aid:(lipid+extract) ratio.

The variables were studied at three main levels, having *α* as ± 1.682 and three replicates at the central points. Table I presents the levels (values) of coded and uncoded variables, and Table II the resulting experimental design. The total concentration and retention of marker content in the proliposomes, *X*_p_, *A*_w_, and product recovery (*R*_ec_), were the experimental responses analyzed.

**Table I:** Uncoded Variables and Their Respective Values

Coded variables	Uncoded variables	Levels				
		− 1.682	− 1	0	+ 1	+ 1.682
A	Lipid concentration (%)	2.0	4.0	7.0	10.0	12.0
B	Extract concentration (%)	0.5	1.5	3.0	4.5	5.5
C	Drying aid:(lipid+extract) ratio	0.86	1.0	1.2	1.4	1.54

**Table II:** Nonrandomized Central Composite Design (CCD) Showing Levels of Coded Variables Used in Proliposome Preparation

Formulation	Codes variables		
	A^a^	B^b^	C^c^
F1	− 1.000	− 1.000	− 1.000
F2	1.000	− 1.000	− 1.000
F3	− 1.000	1.000	− 1.000
F4	1.000	1.000	− 1.000
F5	− 1.000	− 1.000	1.000
F6	1.000	− 1.000	1.000
F7	− 1.000	1.000	1.000
F8	1.000	1.000	1.000
F9	− 1.682	0.000	0.000
F10	1.682	0.000	0.000
F11	0.000	− 1.682	0.000
F12	0.000	1.682	0.000
F13	0.000	0.000	− 1.682
F14	0.000	0.000	1.682
F15	0.000	0.000	0.000
F16	0.000	0.000	0.000
F17	0.000	0.0000.000	0.000

*w.b* wet basis, *d.b* dry basis

^*a*^Lipid concentration (% w/w, w.b.)

^*b*^Extract concentration (% w/w, w.b.)

^*c*^Drying aid:(lipid+extract) ratio (% w/w, d.b.)

This design allows the determination of linear, quadratic, and interaction effects of variables, expressed by the following model (Eq. 1), which can be fitted to experimental data by regression analysis.

1 $$ {Y}_i={a}_0+{a}_1.{X}_1+{a}_2.{X}_2+{a}_3.{X}_3+{a}_{11}.{X}_1^2+{a}_{22}.{X}_2^2+{a}_{33}.{X}_3^2+{a}_{12}.{X}_1.{X}_2+{a}_{13}.{X}_1.{X}_3+{a}_{23}.{X}_2.{X}_3+\varepsilon $$

where *Y*_i_ is the expected response associated with the combination factors, a_0_ to a_33_ are the regression coefficients, *X*_1_ to *X*_3_ denote the factors, and ɛ represents the experimental error. The statistical significance of the effects was tested by analysis of variance (ANOVA).

The statistical significance of the linear, quadratic, and interaction effects of the investigated variables on selected proliposome properties (*X*_p_, *A*_w_, total marker retention, relative marker content, and *R*_ec_) was assessed through ANOVA and regression analysis using the software Statistica^®^ 13.0 (StatSoft Inc., USA).

##### Optimization of Product Quality (Desirability Approach)

The regression models fitted to experimental results allowed the determination of the best condition for proliposome production, by using the multi-response optimization (desirability approach). Desirability values for responses evaluated showed significant dependence on composition variables studied. By using the desirability function in the Statistica^®^ 13.0 software, respective values of each coded variable to reach the desired outcomes were established. Fresh proliposome batch was prepared at the optimum conditions determined, and its properties were compared with those estimated by the regression model. The differences were reported as percentile relative error.

#### Proliposomes Properties and Drying Performance

*X*_p_, *A*_w_, total marker content, marker retention percentage, and zeta potential (ZP) and size distribution of the redispersed powder were the properties used for proliposome characterization. Spray drying performance was evaluated by determination of the *R*_ec_, being the initial solid content/powder production ratio. The experimental methods used are presented as follows:

##### Moisture Content (*X*_p_) and Water Activity (*A*_w_)

*X*_p_ of the SDP was determined by gravimetric method in a moisture analyzer Sartorius MA35 (Goettingen, Germany). *A*_w_ was measured in an AquaLab 4TEV instrument (Decagon Devices Inc., Pullman, WA) at 25°C, using the dew point sensor. Results are expressed as mean and standard deviation of triplicate measurements.

##### Concentration and Retention of Marker Compounds in Proliposomes

The concentration of main rosemary polyphenols in SDP was evaluated by the HPLC-DAD method described in the “Quantification of Polyphenol Markers in the Freeze-Dried Rosemary Extract” section. The retention of marker polyphenols in the SDP was calculated as the concentration of each compound in the proliposome powder (*Q*_f_) relative to the original amount added to the liposome composition (*Q*_i_) by using Eq. (2). Total content of marker in bulk quantity was determined as concentration (w/w) of each compound in any taken sample of proliposome powder, results being mean and standard deviation of triplicate determinations.

2 $$ \mathrm{Retention}\ \left(\%\right)={Q}_f/{Q}_i\cdot 100 $$

##### Proliposome Redispersity, Particle Size, Polydispersity Index, and Zeta Potential

The capability of the powdered product to spontaneously form liposome vesicles was evaluated. Samples of SDP were redispersed in purified water at the same solid concentration (10.9% w/w) of the initial liposomal composition feed to the spray dryer. The mixture was placed under mild agitation for 60 min using a magnetic stirrer (IKA Werke mod. RT 15, Germany).

The particle size, polydispersity index (PDI), and ZP of the reconstituted composition were measured in a Zetasizer (Malvern Nano ZS90, UK) using the principle of dynamic light scattering (DLS) and compared with the values obtained for initial liposome formulations.

##### Product Recovery from Spray Drying

The spray drying conditions were monitored during drying runs including the inlet and outlet spray drying temperatures, environment/room temperature, and relative humidity, to evaluate the system performance. The *R*_ec_ during drying was assumed as a measure of system performance. *R*_ec_ was determined by mass balance in the system, according to Eq. (3), as the percentage amount of proliposome collected from the cyclone relative to solid content of feed formulation (52).

3 $$ {R}_{\mathrm{EC}}=\frac{M_{\mathrm{c}}\left(1={X}_{\mathrm{P}}\right)}{W_{\mathrm{s}}\cdot {C}_{\mathrm{s}}\cdot T}\cdot 100 $$

where *R*_ec_, product recovery (%); *M*_C_, mass of collected proliposome (g); *X*_p_, product moisture content (g); *W*_s_, liquid liposomal composition feed rate (g/min); *C*_S_, solid content of the feed liposomal composition (g); and *T*, process time (min).

### Biological Activities

#### Antioxidant Assay

The antioxidant activity of the LE and optimized SDP was determined by the DPPH^•^ scavenging method (53). In the presence of an antioxidant molecule, the DPPH^•^ is reduced due to its capability of accepting a hydrogen atom supplied by the antioxidant compound. The reduction might be monitored by measuring the concentration-dependent decrease in absorbance at 517 nm, observable as color change from violet to pale yellow. LE and SDP samples were evaluated at 2.8–55.8 μg/mL (LE basis). One milliliter of 0.1 M acetate buffer (pH 5.5), 1 mL of ethanol, and 0.5 mL of 250 μm ethanolic solution of DPPH^•^ were mixed in a test tube, to which 10 μL of the samples under study was added. The absorbance of the solution was measured after 30 min at room temperature. A blank solution was prepared from the reaction mixture without DPPH^•^ solution. Quercetin (0.4–3.0 μg/mL) and the synthetic antioxidants, BHT (5.0–50.0 μg/mL) and butylated hydroxyanisole (BHA) (1.0–10.0 μg/mL), were used as the reference antioxidants (positive controls). Results were expressed as IC_50_, the sample concentration in μg/mL required to reduce 50% of the DPPH^•^ free radicals added to the reaction medium, and inhibition percentage. All determinations were performed in triplicate.

#### Antifungal Assay

In order to highlight the potential uses of the product as a preservative or antimicrobial agent for pharmaceutical, nutraceutical, and cosmetic products, we decided to evaluate the antifungal activity of the optimal SDP and compare with that of the LE, using a *Candida albicans* strain (ATCC1023) as a model. Stock solutions of pure LE and SDP at 10 mg/mL (LE basis) were prepared in 50% methanol and sonicated for 60 min in an ultrasonic bath and thereafter maintained under mild agitation for 30 min using a magnetic stirrer. A second SDP solution was prepared by using only mild agitation on the magnetic stirrer for 90 min. The determination of MIC and MFC was performed by the broth dilution technique according to the guidelines of the Clinical and Laboratory Standard Institute (54) with slight modifications. Briefly, *Candida albicans* was streaked on Sabouraud dextrose agar and incubated for 24 h at 35°C. Five colonies of approximately 1 mm diameter were picked and suspended in 5 mL sterile 0.9% w/v saline solution. The resulting suspension was vortexed for 15 s, and the cell density was spectrophotometrically adjusted to 0.5 McFarland standard at 530 nm wavelength in 0.9% w/v saline solution, resulting in a suspension containing 1–5 × 106 CFU/mL. A working suspension of 1.5 × 103 cells per mL was made in Sabouraud dextrose broth by 1:100 dilution followed by 1:20 dilution. Exactly 100 μL of the final fungal suspension was added to each well containing 100 μL of a doubling diluted test sample. MIC of samples were determined with final fungal density in each well of 0.75 × 103 cells per mL while the final concentration of test samples ranged from 2500 down to 4.883 μg/mL for both pure LE and SDP (LE basis); 1250 to 2.441 μg/mL for ROA; 250 to 0.488 μg/mL for terbinafine, used as positive control; 12.5 to 0.024% for methanol, as vehicle control; and 0.9% w/v saline solution, as negative control. The plates were incubated for 48 h at 35°C after which it was visually examined for the presence or otherwise of fungal growth. Confirmatory test was carried out by adding 20 μL of 0.02% resazurin to each well and further incubating for 1 h at 35°C. Presence of fungal growth was indicated by a change from bluish-purple to pink color, where the bluish-purple color indicates the absence of fungal growth. The MIC was defined as the lowest concentration able to inhibit any visible fungal growth. Ten microliters of the broth from MIC well was then incubated on Sabouraud dextrose agar at 35°C for 24 h for the determination of MFC, the lowest concentration able to kill 100% of the yeasts. Assays were made in triplicates with duplicate controls.

## RESULTS AND DISCUSSION

### Characterization of the Lyophilized Extract of Rosemary

Since the biological activity of rosemary is associated with its major polyphenols – mainly ROA, CAR, CNA, and mildly to CFA (12,55,56), these compounds were selected as chemical markers and quantified in the LE to serve as a baseline in the determination of their concentration and retention in the SDP. Table III presents the experimental values of the concentration of ROA, CAR, CNA, and CFA in the original LE.

**Table III:** Concentration of Polyphenol Markers in Lyophilized Extract of Rosemary

	Concentration of marker polyphenols (% w/w)			
	CFA^a^	ROA^b^	CAR^c^	CNA^d^
Lyophilized extract	0.06 ± 0.005	4.38 ± 0.02	3.69 ± 0.06	3.37 ± 0.06

^*a*^Caffeic acid

^*b*^Rosmarinic acid

^*c*^Carnosol

^*d*^Carnosic acid

### Proliposome Properties and Drying Performance

Table IV presents the results of proliposome properties and *R*_ec_, for all experimental runs carried out (see Table II). Regression analyses were applied to the experimental data to find the statistically significant effects of composition variables on the responses evaluated. Hence, the linear, quadratic, and interaction regression coefficients and their statistical significance at different levels were derived (Table V). The ANOVA and effect estimates were based on assumptions of normal and independent residual distribution, with mean zero and constant variance (57).

**Table IV:** Physicochemical Properties of Spray-Dried Proliposomes and Product Recovery (R_ec_) According to DoE

Exp. runs	*X*_p_ (−)	*A*_w_ (−)	Concentration of marker compounds			Retention of marker compounds			
			ROA^a^ (mg/100 g)	CAR^b^ (mg/100 g)	CNA^c^ (mg/100 g)	ROA^a^ (%)	CAR^b^ (%)	CNA^c^ (%)	R_ec_ (%)
F1	2.92 ± 1.03	0.401 ± 0.004	579.9 ± 16.6	443.7 ± 9.0	355.7 ± 8.0	97.2 ± 2.8	88.1 ± 1.8	77.4 ± 1.7	38.6
F2	2.68 ± 0.90	0.377 ± 0.006	245.9 ± 2.2	244.2 ± 5.8	180.2 ± 2.0	86.1 ± 0.8	101.3 ± 2.4	82.0 ± 0.9	45.8
F3	2.06 ± 0.02	0.404 ± 0.006	1191.6 ± 25.8	814.1 ± 19.5	634.4 ± 4.2	102.9 ± 2.2	83.2 ± 2.0	71.1 ± 0.5	38.2
F4	3.90 ± 0.03	0.453 ± 0.006	664.2 ± 6.8	479.9 ± 6.0	406.7 ± 2.6	97.8 ± 1.0	83.7 ± 1.0	77.8 ± 0.5	28.5
F5	2.71 ± 1.07	0.365 ± 0.009	481.9 ± 3.4	432.6 ± 6.5	432.6 ± 6.5	432.6±6.5	103.0 ± 1.6	84.0 ± 0.9	43.0
F6	2.50 ± 0.78	0.472 ± 0.003	215.1 ± 4.6	193.9 ± 4.9	168.3 ± 6.4	90.5 ± 1.9	96.6 ± 2.4	91.9 ± 3.5	40.9
F7	1.98 ± 0.04	0.430 ± 0.004	975.1 ± 11.3	611.8 ± 7.0	551.5 ± 2.0	101.0 ± 1.2	75.1 ± 0.9	74.2 ± 0.3	40.7
F8	2.06 ± 0.04	0.349 ± 0.002	549.3 ± 3.9	395.1 ± 8.3	371.8 ± 6.6	97.1 ± 0.7	82.7 ± 1.7	85.3 ± 1.5	28.1
F9	2.05 ± 0.00	0.301 ± 0.004	1281.9 ± 18.1	630.8 ± 3.9	614.1 ± 9.9	106.4 ± 1.5	62.0 ± 0.4	66.2 ± 1.1	34.3
F10	3.12 ± 0.22	0.397 ± 0.007	369.0 ± 3.1	309.3 ± 4.5	265.0 ± 1.5	93.0 ± 0.8	92.4 ± 1.3	86.8 ± 0.5	20.1
F11	3.04 ± 0.17	0.402 ± 0.004	80.9.0 ± 3.3	150.7 ± 0.9	81.7 ± 2.2	63.8 ± 2.6	104.6 ± 0.9	83.6 ± 2.2	24.1
F12	1.96 ± 0.01	0.370 ± 0.004	916.0 ± 16.0	546.3 ± 3.9	487.0 ± 5.2	104.4 ± 1.8	73.8 ± 0.5	72.1 ± 0.8	29.5
F13	1.95 ± 0.04	0.429 ± 0.015	699.6 ± 5.1	519.5 ± 4.8	459.6 ± 5.5	99.3 ± 0.1	87.4 ± 0.1	84.7 ± 1.0	39.0
F14	2.50 ± 0.19	0.411 ± 0.015	510.1 ± 12.0	368.7 ± 4.8	343.8 ± 7.3	98.5 ± 2.3	84.4 ± 1.1	86.3 ± 1.8	40.1
F15	1.83 ± 0.13	0.372 ± 0.020	585.4 ± 6.5	425.9 ± .3	364.1 ± 7.0	98.1 ± 1.1	88.5 ± 1.4	79.2 ± 1.5	33.5
F16	1.94 ± 0.07	0.370 ± 0.014	580.9 ± 7.2	465.6 ± 6.2	369.3 ± 2.6	97.3 ± 1.2	88.4 ± 1.2	80.4 ± 0.6	32.5
F17	1.77 ± 0.16	0.395 ± 0.00	578.8 ± 6.0	441.8 ± 3.3	381.3 ± 2.5	97.0 ± 1.0	87.7 ± 0.7	81.0 ± 0.5	32.7

*R*_*ec*_ product (SDP) recovery from spray drying process, *X*_p_ moisture content, *A*_*w*_ water activity

^*a*^Rosmarinic acid

^*b*^Carnosol

^*c*^Carnosic acid

**Table V:** Regression Coefficients and Their Statistical Significance Levels for Product Properties and Product Recovery (R_ec_)

Input factors (lone/interacting)	*X*_p_ (−)	*A*_w_ (−)	Concentration of marker compounds			Retention of marker compounds			R_ec_ (%)
			ROA^a^ (mg/100 g)	CAR^b^ (mg/100 g)	CNA^c^ (mg/100 g)	ROA^a^ (%)	CAR^b^ (%)	CNA^c^ (%)	
*a0*—mean/interc.	1.837*	0.402*	116.776*	88.472*	73.005*	97.342*	88.284*	80.157*	32.451*
*a1*—A_(L)_	0.239***	− 0.001	− 45.242*	− 22.403*	− 19.385*	− 3.587***	4.827***	4.741*	− 3.011
*a11*—A_(Q)_	0.290***	0.009	15.738*	3.249	5.187*	1.268	− 4.116	− 1.192	− 0.479
*a2*—B_(L)_	− 0.193	− 0.008	47.765*	24.194*	23.725*	7.058*	− 12.941*	− 3.383*	− 1.744
*a22*—B_(Q)_	0.260***	0.004	− 7.387***	− 5.343**	− 5.788*	− 4.261**	6.494***	− 0.715	− 0.624
*a3*—C_(L)_	− 0.101	0.000	− 11.404*	− 8.819*	− 5.249*	0.013	− 0.292	2.175**	0.249
*a33*—C_(Q)_	0.161	0.023**	0.136	1.418	2.513**	0.983	− 1.050	1.994**	3.877***
*a12*—A_(L)_ × B_(L)_	0.296***	0.023**	− 8.809***	− 2.817	− 1.958***	1.061	0.162	0.674	− 3.408
*a13*—A_(L)_ × C_(L)_	− 0.216	− 0.015	4.220	1.963	1.748***	0.710	− 1.565	0.965	− 1.515
*a23*—B_(L)_ × C_(L)_	− 0.192	0.025**	− 5.070	− 5.643***	− 1.801***	− 0.832	− 2.424	− 0.740	0.321
Adj. *R*^2^	0.751	0.824	0.967	0.951	*0.992*	0.744	0.720	0.853	0.645

*R*_*ec*_ product (SDP) recovery from spray drying process, *X*_*p*_ moisture content, *A*_*w*_ water activity,* a*_0_ to *a*_23_ regression coefficients

*Effect significant at *p* ≤ 0.01; **effect significant at *p* ≤ 0.05; ***effect significant at *p* ≤ 0.1

*A*, Lipid concentration (% w/w); *B*, extract concentration (% w/w); *C*, drying aid:(lipid+extract) ratio

^*a*^Rosmarinic acid

^*b*^Carnosol

^*c*^Carnosic acid

#### Moisture Content and Water Activity

*X*_p_ of powder samples provides information regarding efficiency of solvent removal during drying and can be linked to their physicochemical stability, solubility, morphology, and flowability. The SDP showed very low values of *X*_p_, in the range 1.7 ± 0.14%–2.5 ± 0.23%, evidencing a slight effect of composition variables. *A*_w_, being a measure of the energy state of water present in a system, is a property independent of sample quantity. The *A*_w_ has significant effect on several degradation reactions such as lipid oxidation and nonenzymatic browning. Values lower than 0.5 are usually recommended to avoid microbial growth, guaranteeing the microbiologic product stability (58). However, the lipid oxidation shows a minimum in the *A*_w_ range of 0.2 to 0.35 and increase outside this range (59). In this work, the values of *A*_w_ were in the range of 0.301 to 0.472, above the lower limit value and slightly above the upper value, guaranteeing the low rates of lipid oxidation of the powdered proliposomes.

Therefore, it can be partially concluded that the drying condition and composition proportions of the liposomal formulation used are suitable for preparing products having potential stability to intra-matrix chemical reactions and microbial proliferation.

*X*_p_ and *A*_w_ of SDP are expected to be linked to drying conditions and formulation composition, since it promotes changes in water binding and dissociation. The regression analyses (Table V) show that *X*_p_ is slightly influenced by the linear and quadratic effects of the variable lipid concentration (A), quadratic effect of the LE concentration (B), and of interaction A × B (*p* ≤ 0.1). The drying aid:(lipid+extract) ratio did not show statistical significance on *X*_p_. However, the observed effects on *X*_p_ were not relevant from an engineering point of view, since the changes in *X*_p_ were small (perhaps due to the identical spray drying condition used).

On the other hand, the influence of the composition variables investigated was more pronounced on *A*_w_. The regression analyses presented in Table V indicate significant effects of the drying aid:(lipid+extract) ratio (quadratic effect), as well as of interaction between lipid *vs* extract concentration and extract *vs* drying aid:(lipid+extract) ratio (A × B and B × C), at *p* ≤ 0.05. These results are expected since the water binding capacity of a dried powder is directly correlated to its composition and structure. Indeed, the SDP structure and propensity for water absorption is intrinsically linked to composition variables investigated. For example, although lactose has been previously used as drying aid in lipid systems encapsulating polyphenols (47), it has been reported that lactose monohydrate loses its water of hydration at 100°C, the drying temperature used in this study (60).

#### Concentration and Retention of Marker Compounds in Proliposomes

The content of major rosemary polyphenols in the SDP is highly linked to the composition variables investigated, since all factors in high or small degree affect the relative quantity of bioactive compounds added to the original liposomal composition. Following logical reasoning, the amount of ROA, CAR, and CNA is positively correlated with the amount of LE added to the original liposomal composition and conversely with the lipid and/or drying aid concentration.

**Fig. 1:**
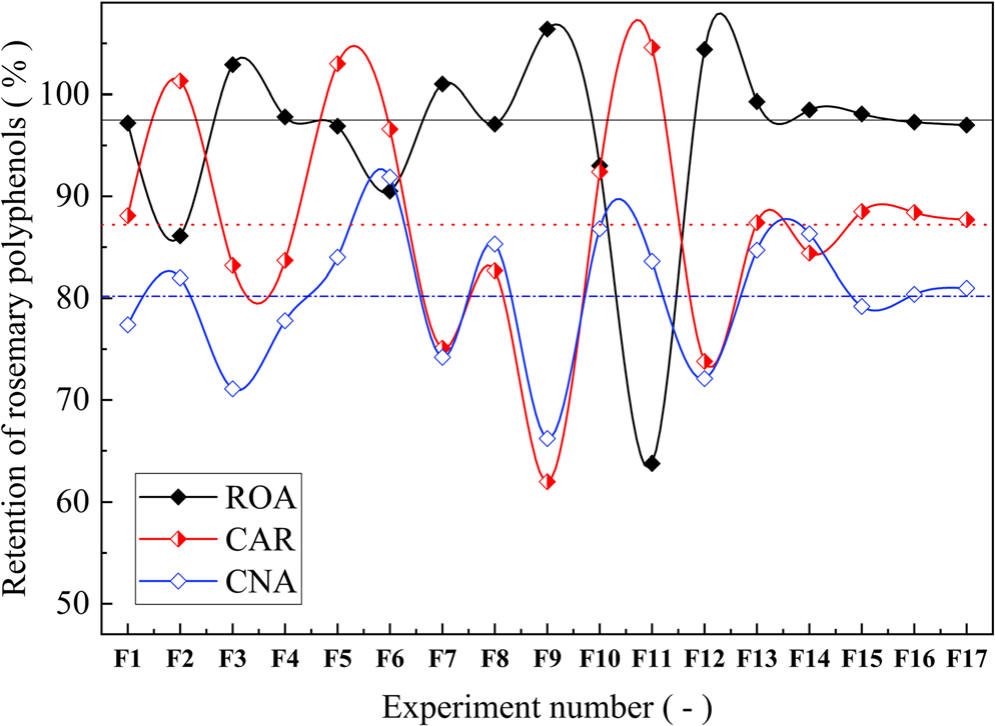
Results of the retention of ROA, CAR, and CNA for all experimental runs

On the other hand, the retention of the bioactive marker compounds in the SDP correlates with composition variables in a more complex way. Figure 1 shows a comparison between the with composition variables in a more complex way. Figure 1 shows a comparison between the retention efficiency of ROA, CAR, and CNA in the SDP. As can be seen in Fig. 1, the percentage retention of ROA was higher than the ones observed for CAR and CNA, with average values of: ROA = 95.7 ± 9.5%, CAR = 87.2 ± 10.9%, and CNA = 80.2 ± 6.6%. Interestingly, the effects of composition variables on retention of CAR and CNA showed similar trends, while ROA shows an opposite behavior for most of the experimental runs. These behaviors can be linked with the intrinsic chemical properties of the specific marker compound (ROA, CAR, or CNA). For example, ROA is a more hydrophilic compound (log P ~1.1 – 1.8), while the diterpenes CAR and CNA are liposoluble (log P ~4.1 – 4.8). Hence, these compounds are partitioned in the aqueous and lipid phases of the encapsulating composition in different ways, affecting their retention efficiency in the SDP. Moreover, CNA is relatively unstable, mainly in solvent, and the air might induce its degradation reaction; CAR being one of its degradation products (61). ROA, on the other hand, is relatively more stable than CAR and CNA (12).

The retention extremes observed in Fig. 1 are in agreement with the lipophilicity of each compound, i.e., the more lipophilic compounds (CAR and CNA) exhibited higher retention values at F11 (highest lipid concentration), while ROA (the less hydrophobic compound) showed better retention at F9 (lowest lipid concentration), and *vice versa.* The method for SDP preparation here presented could also be used for simultaneous encapsulation of multi-constituent materials (natural or synthetic), having varied polarity.

**Fig. 2:**
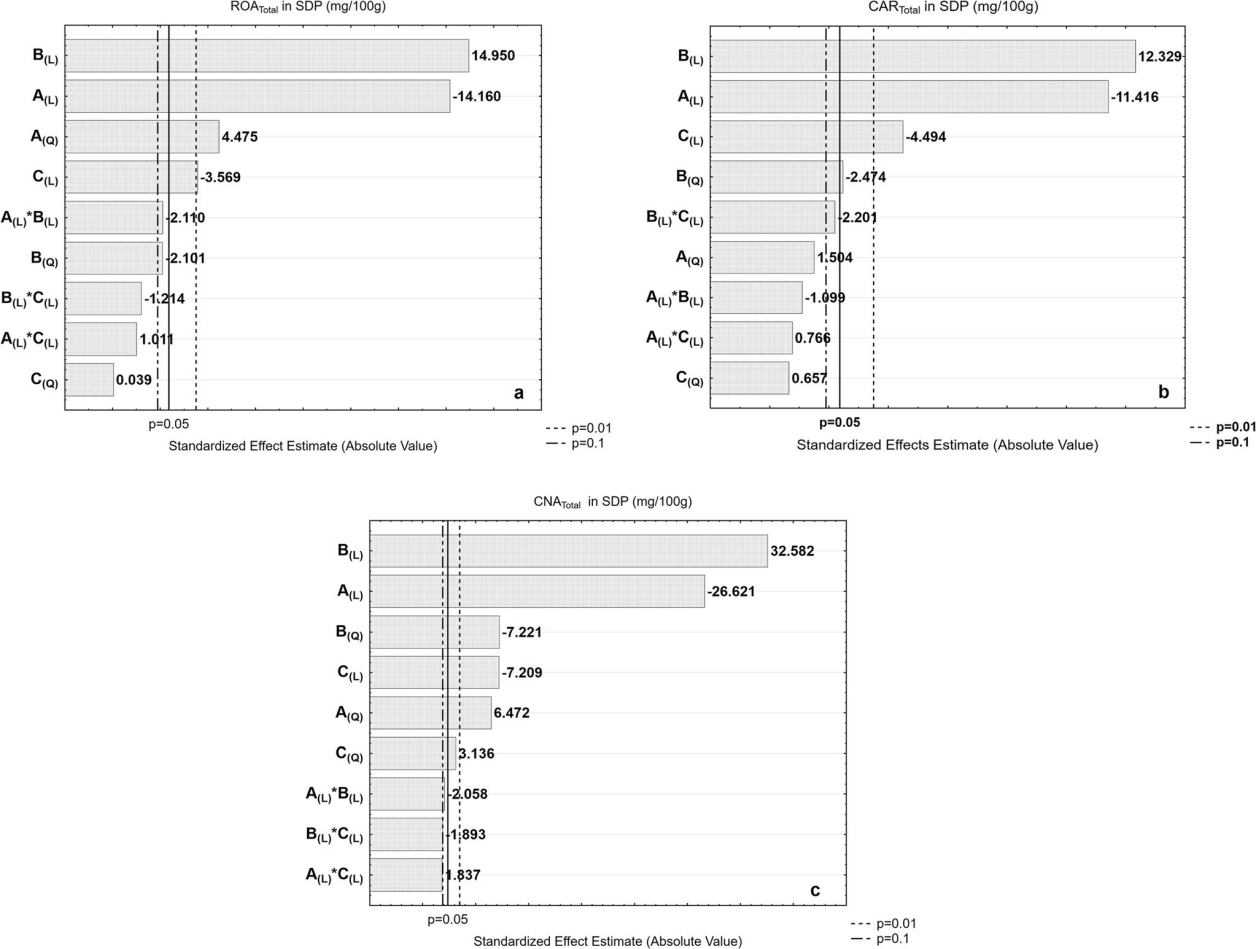
Standardized Pareto charts of studied variables’ effects, respectively, on total content of **a** ROA, **b** CAR, and **c** CNA in the SDP

**Fig. 3:**
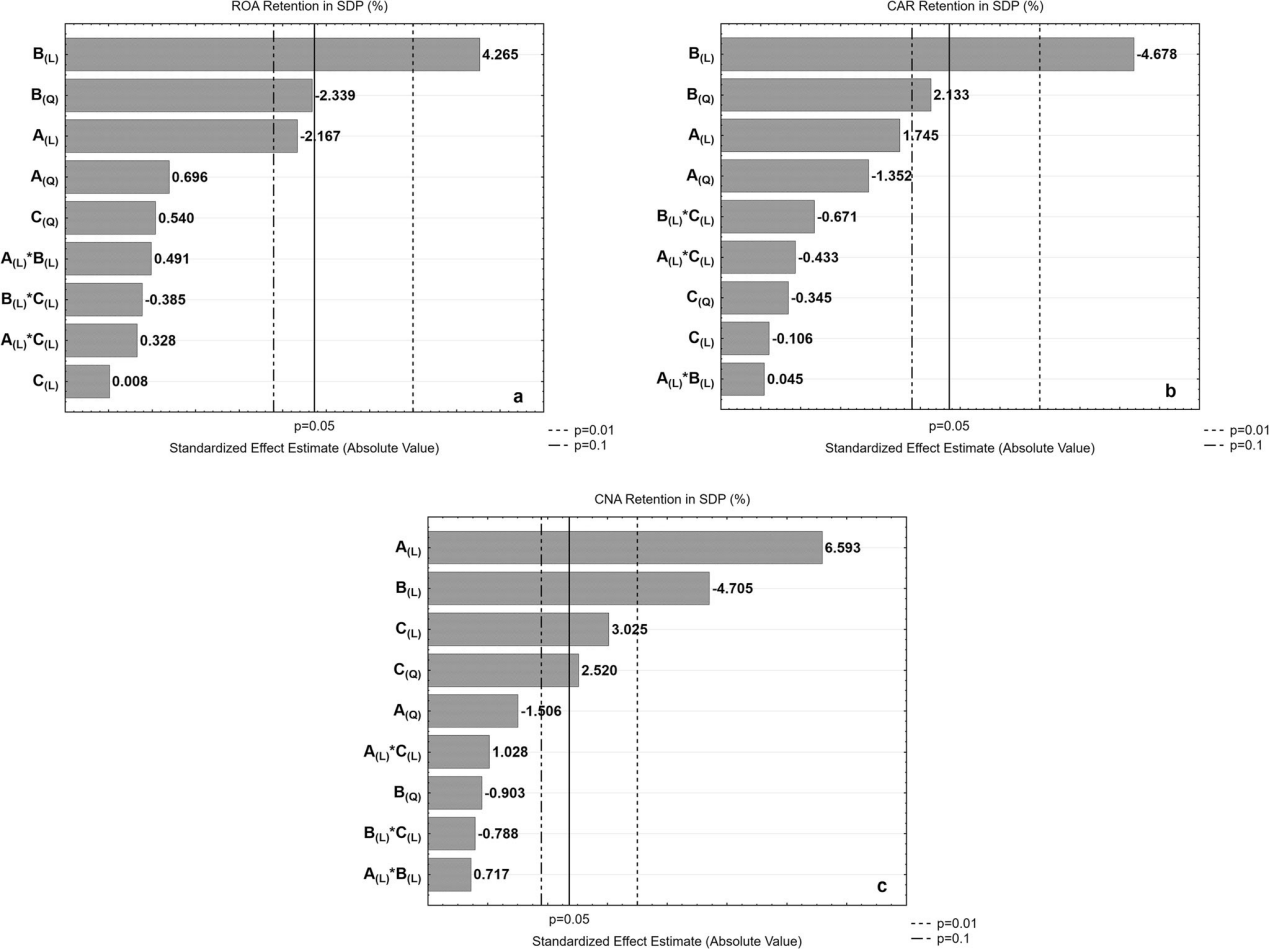
Standardized Pareto charts of studied variables’ effects, respectively, on retention of **a** ROA, **b** CAR, and **c** CNA in SDP

The regression analysis performed for concentration and retention of ROA, CAR, and CNA (see Table V) describes properly the behavior physically expected and shows an acceptable agreement with the experimental data (0.951 ≤ *R*^2^ ≤ 0.992 and 0.720 ≤ *R*^2^ ≤ 0.853), respectively. A convenient way to view the magnitude of the effects of each factor over the dependent variables is through the construction of Pareto chart of the standardized effects. Figures 2 and 3 show the resulting Pareto Charts of the effects of investigated factors for concentration and retention of ROA, CAR, and CNA in the formed SDP, respectively (A: lipid concentration; B: LE concentration; and C: drying aid:(lipid+extract) ratio). The increase in extract concentration was positive for the retention of ROA, but detrimental to the retention of CAR and CNA (effects highly significant, *p* ≤ 0.01). These behaviors are also linked to compound stability and lipophilicity, as discussed beforehand. Zhang *et al.* (12) suggested a first-order, concentration-dependent degradation pattern for CAR, similar to what is observed for the compound in this system during processing and possibly giving rise to decreased retention as concentration increases. Its relatively higher lipophilicity also suggests favored partitioning into the lipidic wall of the proliposomes rather than the aqueous core. Hence, higher ratio of extract to lipid presumably led to greater retention of hydrophilic compounds apparently protected in the aqueous vesicle core rather than lipophilic components which are no longer efficiently encapsulated and therefore exposed to degradation at the vesicle periphery (62).

The linear effect of lipid concentration further demonstrates this relationship, although showing a quasi-significance (*p* ≤ 0.10) for both ROA and CAR retention with negative and positive effects, respectively. Increasing the lipid concentration in the liposomal composition bring benefits for retention of the nonpolar compounds CAR and CNA (positive signals in the higher regression coefficients, a1), but is disadvantageous for the retention of ROA.

Regarding CNA retention, evaluated lone factors ranged from being significant (*p* ≤ 0.05) to highly significant (*p* ≤ 0.01). Lipid and drying aid concentrations showed positive effects on CNA, highly significant (*p* ≤ 0.01), similar to those observed for CAR and in line with their lipophilicity. Notwithstanding, the drying aid:(lipid+extract) ratio showed statistically significant effect only for the retention of CNA (*p* ≤ 0.05), a positive effect. Since the degradation of CAR is concentration dependent as we previously suggested, the reaction is skewed away from buildup of its concentration, hence further degradation of CNA (63). The significant positive effect attributed to drying aid concentration on CNA retention might be due to protective effect offered by lactose molecules, thereby slowing down or preventing degradation of the compound (47,64). Evaluated factors showed no interaction effect on retention of bioactive compounds in the SDP. Integrity of the bioactive compounds was largely preserved exhibiting retention greater than 60.0% of each compound for all SDP batches.

**Fig. 4:**
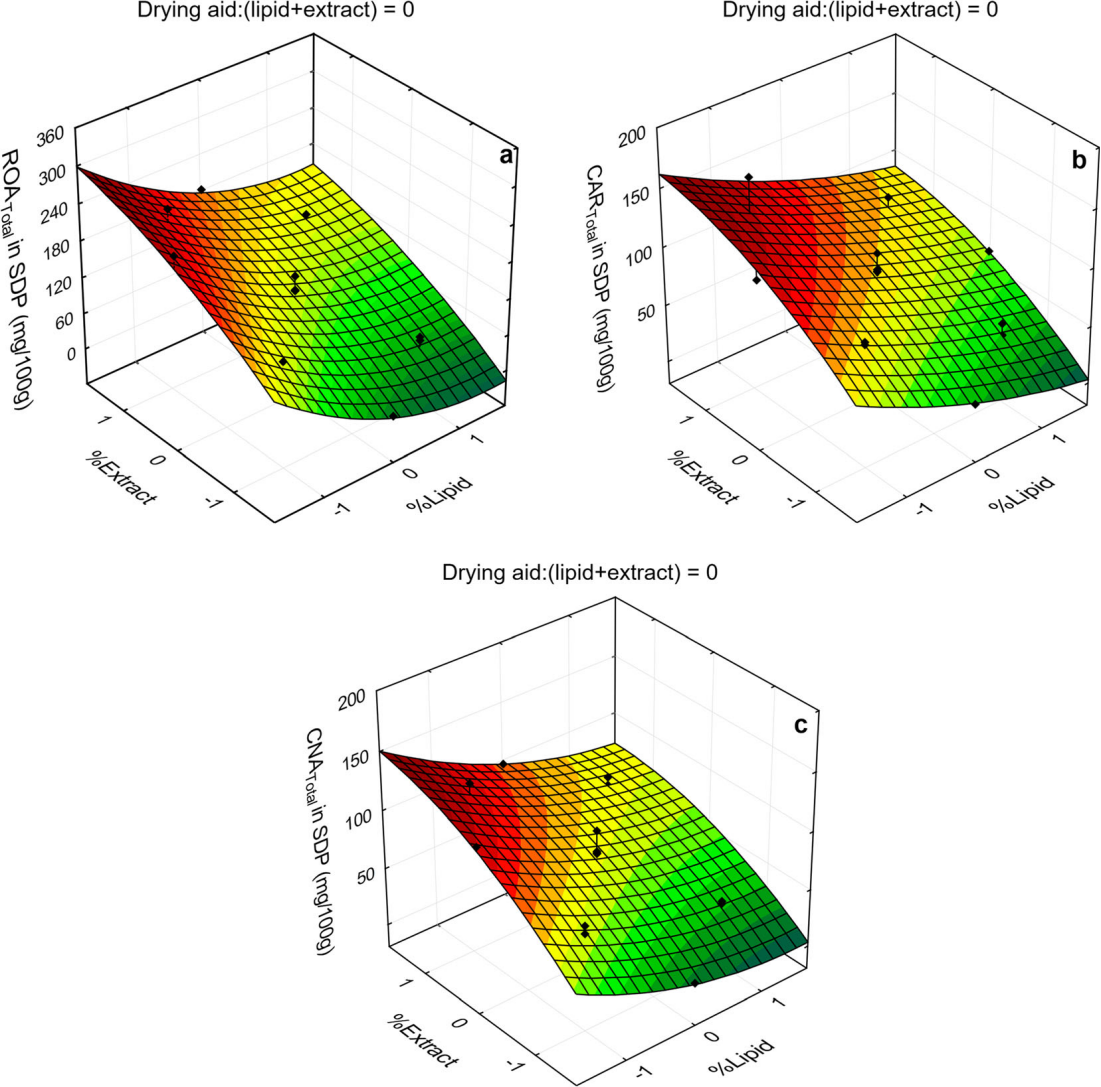
Response surface plots for the total content of ROA, CAR, and CNA in SDP as a function of the significant variables

**Fig. 5:**
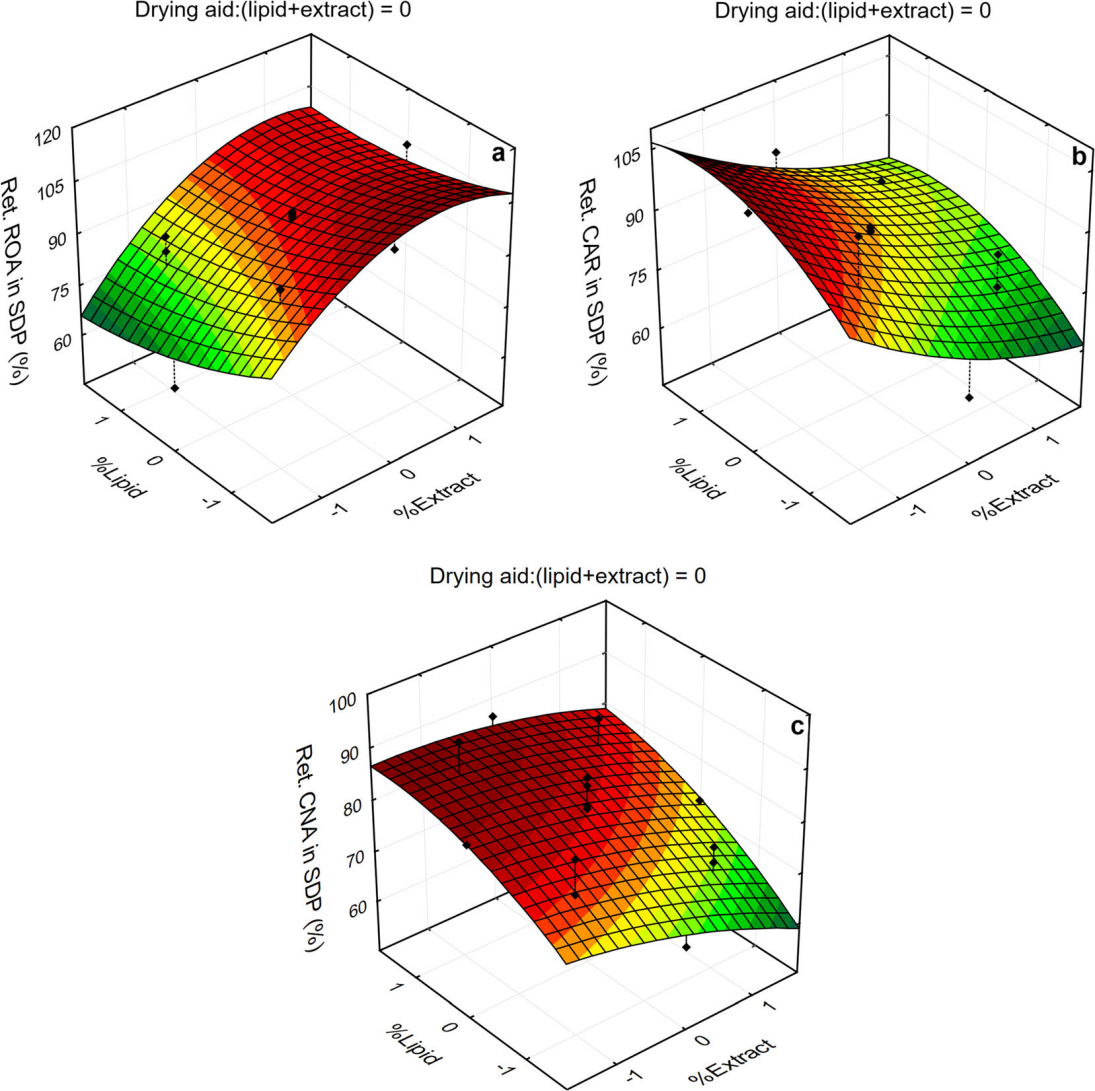
Response surface plots for the retention of ROA, CAR, and CNA in SDP as a function of the significant variables

Figures 4 and 5 present surface response plots showing the effects of the most significant variables (extract and lipid concentrations) on the concentration and retention of ROA, CAR, and CNA in the SDP, respectively. The plots were obtained for the drying aid ratio at midpoint (0.0), which are representative of those obtained at both the lowest (− 1.682) and highest (+ 1.682) drying aid levels. As can be seen in the graphs presented in Fig. 4, the effects of the composition variables on the concentration of ROA, CAR, and CNA in the SDP exhibit high similarity, evidencing the predominance of “dilution” effects on these responses.

Similarly, relationship between studied variables and marker retention response revealed that retention patterns of polyphenol compounds at 0.0 (mean) level of lactose concentration (Fig. 5) are similar to those obtained at − 1.682 (low) and + 1.682 (high) levels. While ROA retention is facilitated at high extract concentration, both CAR and CNA are favorably retained at high lipid concentration and low extract concentration levels.

#### Proliposome Redispersity, Particle Size, Polydispersity Index, and Zeta Potential

A powdered product is essentially referred to as a proliposome if it has the capacity to easily form liposomes when hydrated. Here, the SDP were readily redispersible in water at the same original concentration, promptly forming vesicles loaded with the rosemary polyphenols encapsulated in the dried system. The mean particle size, PDI, and ZP of vesicles resulting from SDP redispersion were compared with the values obtained for initially prepared liquid liposomal formulation (LLF) prior to spray drying (see Table VI). Higher particle sizes were observed from hydrated SDP (HSDP) compared with the corresponding LLF from which it was derived. While particle size ranged between 668 and 3006 nm for LLF, the sizes of vesicles obtained from HSDP ranged between 1478 and 4530 nm. The proliposome method is known to generate multilamellar liposome vesicles (65,66) whose vesicle diameters are usually greater than small unilamellar liposomes; this phenomenon being principally responsible for the increment in vesicle sizes. Also, association of the drying aid (lactose) with the vesicles might also have contributed to the increment in size. Matsumoto (67) discussed the concentration-dependent effect of sugars on ZP of vesicular globules, thought to be brought about by the expanding location of the slipping plane during electrophoretic movement, leading to the formation of a viscous hydration layer on the surface of the globules. This layer apparently appears as part of the vesicle, resulting in higher values during size measurement. No specific pattern of change was observed, and the differences might probably result from process influences. Notwithstanding, in a further assay, the particle size obtained from HSDP was successfully reduced by up to 60% by bath sonication of samples for 60 min, without attendant disruption of vesicle stability (samples used for antifungal test in this study). PDI values were generally ≥ 0.5 and ZP was below − 20 mV for all formulations. While the ZP values indicate a potential thermodynamic stability of both LLF and HSDP, their PDI values can be considered adequate since the formulations developed have not been proposed for parenteral medical uses in which such disparity in particle sizes is unacceptable. Vesicles from HSDP showed higher ZP than those corresponding LLF, perhaps due to the addition of the drying aid to liposomal composition.

**Table VI:** Particle Properties of Liquid Liposome Formulation (LLF) *vs* Hydrated Spray-Dried Proliposome (HSDP) Encapsulating Rosemary Polyphenols (Nonrandomized Central Composite Design)

Formulation runs	Particle diameter (nm)		Polydispersity index (PDI) (−)		Zeta potential (ZP) (mV)	
	LLF	HSDP	LLF	HSDP	LLF	HSDP
F1	1818 ± 350	2531 ± 521	1.00 ± 0.00	0.70 ± 0.13	− 38.4 ± 1.1	− 28.5 ± 1.2
F2	1307 ± 49	1478 ± 94	0.89 ± 0.05	0.96 ± 0.04	− 43.6 ± 0.8	− 24.7 ± 2.1
F3	1045 ± 223	2398 ± 418	1.00 ± 0.00	1.00 ± 0.00	− 34 ± 4.3	− 20.7 ± 1.6
F4	1724 ± 113	4530 ± 774	0.88 ± 0.03	0.34 ± 0.07	− 33.1 ± 2.2	− 24.0 ± 1.8
F5	2909 ± 478	3299 ± 406	1.00 ± 0.00	0.53 ± 0.06	− 35.1 ± 2.7	− 22.7 ± 3.2
F6	836 ± 27	1661 ± 153	0.65 ± 0.09	0.83 ± 0.27	− 37.8 ± 0.9	− 24.4 ± 0.9
F7	874 ± 124	3144 ± 287	1.00 ± 0.00	0.69 ± 0.04	− 27.8 ± 3.1	− 20.6 ± 0.8
F8	1835 ± 202	3150 ± 387	0.92 ± 0.08	0.46 ± 0.04	− 31.4 ± 1.5	− 20.2 ± 0.7
F9	831 ± 130	2878 ± 176	0.88 ± 0.09	0.64 ± 0.14	− 31.6 ± 2.7	− 27.9 ± 2.2
F10	1776 ± 191	1721 ± 309	0.63 ± 0.20	0.83 ± 0.19	− 37.5 ± 0.4	− 25.7 ± 1.0
F11	668 ± 122	1692 ± 254	0.92 ± 0.08	1.00 ± 0.00	− 34.0 ± 1.6	− 26.2 ± 1.0
F12	734 ± 90	2578 ± 330	0.98 ± 0.03	1.00 ± 0.00	− 33.5 ± 2.4	− 28.4 ± 0.4
F13	2371 ± 195	3922 ± 732	0.93 ± 0.13	0.58 ± 0.06	− 35.8 ± 2.6	− 32.2 ± 1.0
F14	1750 ± 180	2872 ± 321	0.86 ± 0.06	0.88 ± 0.21	− 34.2 ± 3.1	− 31.3 ± 1.3
F15	3006 ± 297	4166 ± 308	1.00 ± 0.00	0.97 ± 0.05	− 40.5 ± 2.5	− 29.3 ± 1.6
F16	2541 ± 199	4292 ± 457	1.00 ± 0.00	0.86 ± 0.24	− 33.8 ± 1.5	− 30.9 ± 0.6
F17	2646 ± 408	4064 ± 589	0.61 ± 0.02	0.89 ± 0.12	− 41.7 ± 1.7	− 27.9 ± 1.5

#### Powder Recuperation from Spray Drying Operation

The *R*_ec_ from the spray dryer ranged between 20.1 and 45.8% (Table V). This relatively low percentage might be attributed to losses by elutriation of the fine particles generated during the spray drying, a common occurrence in bench-top spray dryers using cyclone as unique powder collection system. Another cause is the stickiness of a parcel of the atomized product on dryer wall, which has been shown to be dependent on the glass transition temperature of the feed composition and drying temperature used (68). This is really critical during spray drying of lipid compositions due to the low phase transition point of lipid constituents. For example, the Phospholipon 90H has melting temperature between 55 and 67°C (69), with a glass transition temperature below this value. Hence, both the lipid and the extract tended to reduce the glass transition temperature of SDP, while the drying aid increases (data not shown).

In fact, the results of the regression analysis performed for *R*_ec_ (Table V) evidenced a negative tendency of the concentration of the lipid and of rosemary LE added to the liposomal formulation (although not significant statistically), while the effect of the drying aid was positive (*p* ≤ 0.1). *R*_ec_ might be a critical issue, especially in industrial applications where it may be employed as a measure of process efficiency and in the analysis of cost implications (70). Since the proliposome approach to encapsulate rosemary polyphenols and other phytopharmaceuticals holds a great potential for application in a large industrial production scale, it is important to put the *R*_ec_ and process efficiency in perspective during research and developmental stages. Approaches for improvement of *R*_ec_ during spray drying have been discussed elsewhere (46,52,71).

### Determination of the Optimum Condition for Proliposome Preparation by Multi-response Optimization—the Desirability Approach

The multi-response optimization (the desirability approach) was applied to the mathematical models fitted to the experimental results to determine the best formulation composition ratios that will generate SDP with acceptable values for *A*_w_ and *X*_p_, high retention and concentration of polyphenols marker compounds, and adequate powder recovery at the spray drying condition utilized. The optimization procedure was implemented in the software Statistica® 13.0 (StatSoft Inc.), by using predefined ranges of each response. Table VII presents the estimated optimum processing conditions.

**Table VII:** Optimized Processing Conditions for SDP Production, Coded and Uncoded Values

Factor	Coded value	Uncoded value
Lipid concentration (% w/w, w.b.)	− 0.841	4.26
Extract concentration (% w/w, w.b.)	+ 0.841	4.48
Drying aid:(lipid+extract) ratio (% w/w d.b.)	− 1.682	0.86

*w.b* wet basis, *d.b* dry basis

The drying aid:(lipid+extract) ratio was predicted as 0.86, corresponding to 7.55% w/w on wet basis. In order to confirm the validity of the optimization procedure, an additional experiment on wet basis was carried out using the optimum formulation composition ratios determined. Table VIII shows the comparison between experimental and predicted SDP properties obtained at optimum processing conditions. It can be observed that relative errors between the experimental and predicted values showed concurrence for all responses except for the percentage retention of CAR, which was 22% lower than the predicted value. This might be either solely due to the mathematical model used in the optimization of CAR, which presented the lowest *R*^2^ (0.720) or in combination with experimental error incurred during quantification of this compound.

**Table VIII:** Predicted and Experimental Values of Quality Attributes of SDP at Optimum Points

Quality attribute	Experimental value	Predicted value	Relative error (%)
Water activity (−)	0.387 ± 0.012	0.402	− 3.9
Moisture content (% w/w)	2.03 ± 0.14	1.84	9.4
ROA retention (% w/w)	100.0 ± 2.5	97.3	2.7
CAR retention (% w/w)	72.0 ± 6.6	88.3	− 22.7
CNA retention (% w/w)	83.1 ± 4.4	80.2	3.5
ROA content (mg/100 g)	615 + 23	583.9	5.1
CAR content (mg/100 g)	431.0 + 9.5	442.3	− 2.6
CNA content (mg/100 g)	375 + 13	371.7	1.0

*ROA* rosmarinic acid, *CAR* carnosol, *CNA* carnosic acid

### Biological Activities

#### Antioxidant Assay

Results of DPPH^•^ reduction produced by pure LE, optimized SDP, the synthetic antioxidants BHT and BHA, and quercetin are shown in Table IX. BHT and BHA have been used as antioxidants in foods and personal care product/cosmetic ingredient, among other applications. However, deleterious effects to humans linked to them have stimulated the search for viable and safe alternatives (56,72–74). Rosemary extracts are commercially available for use as a natural antioxidant for foods in Europe and the USA and has received GRAS (generally recognized as safe) status, being considered safe and effective (7,75,76). Results here reported (Table IX) showed that LE (IC_50_ = 10.7 μg/mL) and SDP (IC_50_ = 9.2 μg/mL—LE basis) both have superior antioxidant activity compared with BHT (12.5 μg/mL). Antioxidant activity of rosemary polyphenols was observed to be similar to that obtained for quercetin (comparison on ROA, CAR, and CNA concentration basis; see Table III). As shown in Table IX, encapsulation of LE in phospholipid-based proliposome enhanced the antioxidant activity of LE (lower IC_50_). This is in accordance with previous studies showing improved antioxidant activity of natural compounds by complexation and encapsulation (77,78). Pinsuwan *et al.* (79) demonstrated the enhanced antioxidant activity of liposomes encapsulating extract of *Hibiscus sabdariffa* using an *in vitro* skin model. A similar *in vitro* activity enhancement using liposome-based encapsulation of natural products has been reported by other researchers using different methods (78,80). Feng *et al.* evaluated the *in vivo* effect of liposomal encapsulation of chlorogenic acid following oral administration in mice. They observed that administration of free chlorogenic acid and liposome encapsulation significantly decreased the relative liver weight hitherto induced by tetrachloromethane. However, there was significantly higher increase in the activities of antioxidant liver enzymes GSH-Px and T-SOD for animals that received liposome-encapsulated chlorogenic acid compared with free compound (81). These improvements observed have been associated with increase in solubility upon liposome formulation which in turn improves interaction with free radicals (77,82).

**Table IX:** IC_50_ Values and DPPH^•^ Inhibition Capacity of the SDP, Compared With LE, the Synthetic Antioxidants (BHT and BHA), and Quercetin

Sample	IC_50_ (μg/mL)a	Inhibition (%)
LE	10.8 ± 0.3*	89.0 ± 0.1
SDP	9.2 ± 0.2*	83.1 ± 0.9
BHT	12.5 ± 0.6*	88.2 ± 0.2
BHA	3.0 ± 0.2*	85.8 ± 0.8
QCT	1.0 ± 0.1*	86.3 ± 0.9

*LE* lyophilized rosemary extract, *SDP* spray-dried proliposome, *BHT* butylated hydroxytoluene, *BHA* butylated hydroxyanisole, *QCT* Quercetin

^*a*^Antioxidant activity by the DPPH^•^ method, expressed as IC_50_

*p < 0.05 is the statistical difference determined by one-way ANOVA followed by Tukey’s post-hoc test

#### Antifungal Assay

Several studies report potent antimicrobial activity of rosemary extracts obtained by different methods (7,83–85) and suggested to be dependent on polyphenolic compounds (19). Hence, it was decided to investigate the antimicrobial activity of the optimal SDP and the LE, using as a model a strain of *Candida albicans* (ATCC1023), aiming to emphasize possible SDP applications in pharmaceutical or food compositions. Table X shows the experimental results of MIC and MFC values against *C. albicans* for pure LE, HSDP, ROA, and terbinafine (positive control) using the microdilution method. ROA, the most abundant polyphenol in the extract, was used as an internal control to detect if it contributes to the antifungal activity while methanol was included as a solvent control. All test samples showed antifungal activity within concentration ranges tested except ROA and methanol. HSDP samples showed similar level of activity to pure LE, indicating that bioactive compounds remain intact and successfully retained during proliposome preparation.

**Table X:** *In Vitro* Sensitivity of *Candida albicans* to Rosemary Lyophilized Extract (LE), Hydrated Spray-Dried Proliposome (HSDP), Rosmarinic Acid (ROA) and Terbinafine (Positive Controls), Methanol (Solvent Control) and 0.9 w/v Saline Solution (Negative Controls), Determined by Broth Microdilution Method

Test sample	MIC^c^ (μg/mL)	MFC^d^ (μg/mL)
Pure LE^a^	312.5	1250
HSDP^b^ (sonicated)	312.5	1250
HSDP^b^ (not sonicated)	312.5	1250
Rosmarinic acid	> 1250	> 1250
Terbinafine	≤ 0.4883	0.9766
Methanol	> 12.5%	> 12.5%
0.9 w/v saline solution	na*	na*

^*a*^Lyophilized extract of rosemary

^*b*^Hydrated spray-dried proliposome

^*c*^Minimum inhibitory concentration

^*d*^Minimum fungicidal concentration

*na “no activity” observed against tested microorganism

It was observed that HSDP sample sonication did not have any effect on its antifungal activity. This suggests that prepared proliposomes may be used as an antifungal agent without any complicated process of redispersion. This is particularly desirable since prepared products are considered to enhance dispersibility in aqueous medium and improve stability of bioactive compounds compared with pure extract. LE and HSDP samples gave MIC of 312.5 μg/mL, well below ≤ 1000 μg/mL, the value suggested for plant extracts (86,87), and hence, were considered effective against the test microorganism. ROA gave no activity at used concentration range which is several folds higher than its concentration in the extract. This suggests that antifungal activity observed is perhaps due to CAR and CNA, rather than ROA. This is in concordance with previous studies (8,9,88) on antimicrobial activity of rosemary extracts. A closer look at obtained results for retention of bioactive compounds revealed that although both LE and HSDP showed similar MIC values, the concentration (as shown by percentage retention) of CAR and CNA was lower in HSDP by 28.0% and 16.9%, respectively (Table VIII). Since these compounds are here suggested to be responsible for the bioactivity under discussion, it is supposed that HSDP system, in reality, has a higher efficiency for the delivery of these compounds against *C. albicans.* Terbinafine is a broad-spectrum antifungal agent, applicable for both oral and topical routes of administration. Although the MIC obtained for terbinafine appears far lower than those of HSDP, it should be borne in mind that the values quoted for HSDP were based on whole extract. Dilute methanol, used as solvent for samples based on LE gave no activity against *C. albicans*, confirming that antifungal effects observed are due to bioactive compounds present in the extract.

## CONCLUSIONS

In this study, proliposome was shown as a viable system for the encapsulation of rosemary polyphenols through a systematic study of the relationships between composition variables and their effects on desirable responses, guided by experimental design. It was shown that relative concentration and retention of each rosemary polyphenol in SDP is a function of its own polarity and composition variables. Whereas ROA retention is largely dependent on concentration of the extract, values for CAR and CNA are influenced by lipid, extract, and drying aid concentrations. *A*_w_ depends on the drying aid (lactose) concentration while *X*_p_ is only slightly influenced by both lipid and extract concentrations.

Optimization of the processing variables using multi-response analysis was successfully validated. The experimental responses determined at optimum processing condition exhibited a good agreement with the estimated values. The optimized SDP loaded with the rosemary polyphenols showed an enhancement of the antioxidant activity and improved efficacy against the yeast, *C. albicans*, when compared with pure LE. These results furnish strong evidences that prepared SDP, having improved physicochemical properties and superior bioactivity, might be applicable as a natural antioxidant or as a phytopharmaceutical agent in treatment and prevention of several acute/chronic diseases in humans, either singly or as a component of a pharmaceutical dosage form. It might also be used as natural preservative in cosmetics and skin care preparations where yeasts such as *Candida* spp. remain a source of contamination and degradation.
